# Supplementary material for: Teaching an old dog new tricks: serum troponin T as a biomarker in amyotrophic lateral sclerosis
Source: Brain Commun. 2021 Nov 17;3(4):fcab274. doi: 10.1093/braincomms/fcab274 (PMC8728713; doi:10.1093/braincomms/fcab274)
Supplement: fcab274_Supplementary_Data [file fcab274_Supplementary_Data.zip › Supplementary Figure Legend.docx]

**Supplementary Figure Legend**

**Supplementary Figure 1: cTnT and ΔALS-FRSr/month**

Correlation analyses were performed using non-parametric Spearman correlations (r). Curves were drawn by a linear regression model with an interaction term for cTnT in serum by

**(A)** ΔALS-FRSr/month in the whole cohort.

**(B)** ΔALS-FRSr/month and cTnT in slow progressors (ΔALS-FRSr/month ≤1).

**(C)** ΔALS-FRSr/month and cTnT in fast progressors (ΔALS-FRSr/month > 1).
