# Supplementary figures and images for: Teaching an old dog new tricks: serum troponin T as a biomarker in amyotrophic lateral sclerosis
Source: Brain Commun. 2021 Nov 17;3(4):fcab274. doi: 10.1093/braincomms/fcab274 (PMC8728713; doi:10.1093/braincomms/fcab274)

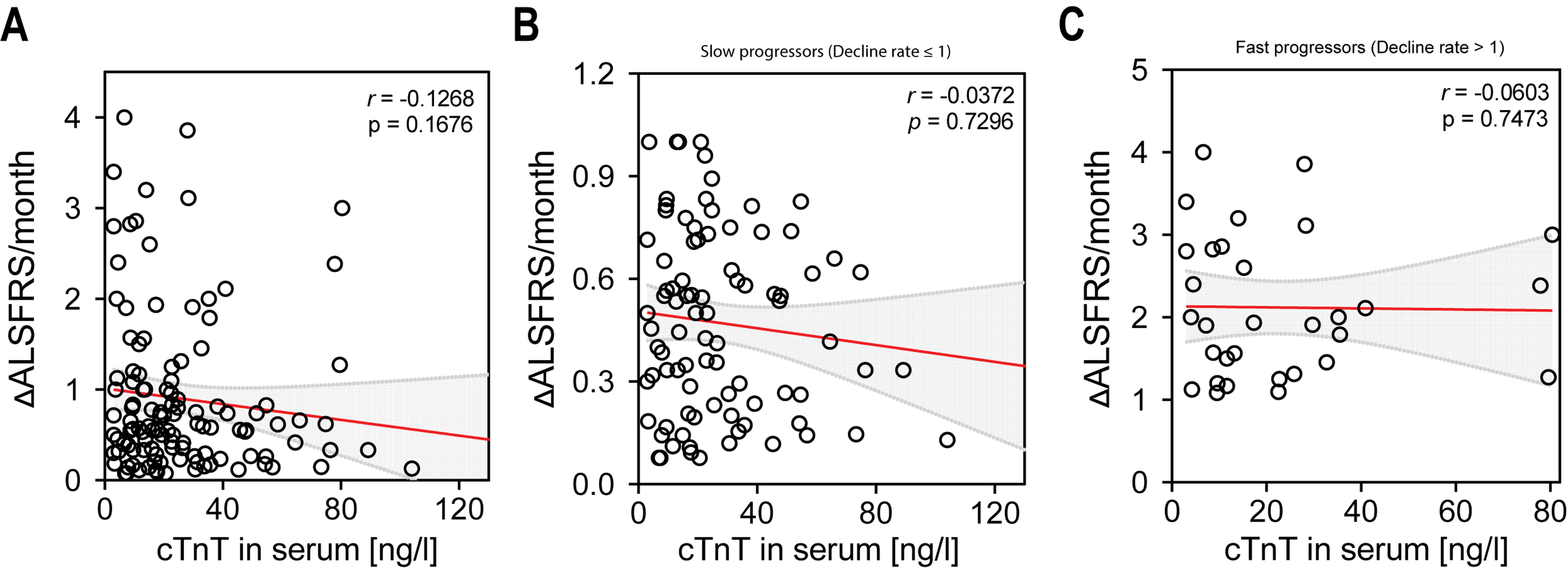

Supplement: fcab274_Supplementary_Data [file fcab274_Supplementary_Data.zip › Supplementary Figure 1.tif]
